# Anxiety Is Associated With DPPIV Alterations in Children With Selective Mutism and Social Anxiety Disorder: A Pilot Study

**DOI:** 10.3389/fpsyt.2021.644553

**Published:** 2021-06-29

**Authors:** Yulia Golub, Valeska Stonawski, Anne C. Plank, Anna Eichler, Oliver Kratz, Regina Waltes, Stephan von Hoersten, Veit Roessner, Christine M. Freitag

**Affiliations:** ^1^Department of Child and Adolescent Psychiatry, Faculty of Medicine, TU Dresden, Dresden, Germany; ^2^Department of Child and Adolescent Psychiatry, Psychosomatics and Psychotherapy, University Hospital Frankfurt, Goethe University, Frankfurt am Main, Germany; ^3^Department of Child and Adolescent Mental Health, University Hospital Erlangen, Friedrich-Alexander University Erlangen-Nürnberg, Erlangen, Germany; ^4^Department of Experimental Therapy and Preclinical Center, Friedrich-Alexander University Erlangen-Nürnberg, Erlangen, Germany

**Keywords:** anxiety, behavioral inhibition, mutism, social anxiety, DPPIV

## Abstract

**Background:** Both selective mutism (SM) and social anxiety disorder (SAD) are severe pediatric anxiety disorders with the common trait of behavioral inhibition (BI). The underlying pathophysiology of these disorders remains poorly understood, however converging evidence suggests that alterations in several peripheral molecular pathways might be involved. In a pilot study, we investigated alterations in plasma molecular markers (dipeptidyl peptidase-4 [DPPIV], interleukin-6 [IL-6], tumor necrosis factor-β [TNF-β] and neuropeptide-Y [NPY]) in children with SM, SAD, and healthy controls, as well as the correlation of these markers to symptom severity.

**Methods:** We included 51 children and adolescents (aged 5–18 years; *n* = 29 girls): *n* = 20 children in the SM-, *n* = 16 in the SAD- and *n* = 15 in the control-group (CG). Peripheral blood samples were analyzed for DPPIV, IL-6, TNF-β, and NPY concentrations. Diverse psychometric measures were used for BI, anxiety, and mutism symptoms.

**Results:** Lower DPPIV-levels were correlated with more anxiety symptoms. However, we could not find a difference in any molecular marker between the patients with SAD and SM in comparison to the CG.

**Conclusion:** DPPIV is proposed as relevant marker for child and adolescent anxiety. Investigating the pathophysiology of SM and SAD focusing on state and trait variables as anxiety or BI might help better understanding the underlying mechanisms of these disorders. Further studies with especially larger cohorts are needed to validate the current pilot-findings.

## Introduction

Both Selective Mutism (SM) and Social Anxiety Disorder (SAD) are severe and debilitating pediatric anxiety disorders often comorbid and closely related to each other. SAD is characterized by marked fearfulness and anxiety in social and performance-related situations, frequently resulting in avoidance, and marked disability. SM is characterized by an inability to speak in specific, particularly unfamiliar social situations despite normal language development and being able to speak correctly in familiar situations (1). Most commonly, SM initially manifests during kindergarten or school, and results in significant social and academic impairment. Along with mute reactions, children with SM may also appear inhibited or frozen and inactive in specific situations (2).

In fact, accumulating evidence suggests that the common trait of behavioral inhibition (BI) contributes to the development of both SAD and SM, with environmental factors stabilizing avoidance behavior (2). BI occurs in response to novelty and potential threat and is associated with autonomic and pituitary-adrenal activation (see for review 3). It is originally defined as an “initial tendency to withdraw, to seek a parent, and to inhibit play and vocalization following encounter with unfamiliar people and events” (3). BI is thought to represent a lower threshold to limbic and sympathetic nervous system arousal (i.e., higher heart rate, reduced heart period variability under stress, increased laryngeal muscle tension). Some authors even conceptualized SAD and SM as different stages in a developmental progression of behaviorally inhibited temperament (4) or suggested that SM represents “the extreme end of a continuum of temperament and social behavior that has a biological basis” (5). According to the etiological model of Johnson and Wintgens (6) intense arousal of the sympathetic nervous system in novel situations during early childhood of SM children may lead to a freezing reaction resulting in reduced confrontations with feared stimuli and thus first shaping and then habituating an avoidance behavior in the form of muteness and inactivity. Though the close link between SM and SAD is widely recognized (5, 7–9), it has to be clarified if the two conditions are only comorbidities or represent two stages on a continuum of a biological trait, sharing the same causative core biological mechanisms.

Both experimental and clinical studies demonstrate an involvement of the immune system in the development of anxiety disorders (10, 11). Patients with anxiety disorders were described to have an increased risk of comorbid neurological, vascular, respiratory, and metabolic conditions (12, 13). Pediatric anxiety disorders were repeatedly found to be associated with atopic disorders, in particular with asthma and allergic rhinitis (14, 15). Several clinical investigations report the peripheral activation of the immune system and the crucial role of cytokines, including, TNF-β and IL-6, in major depressive disorder and chronic anxious states (16–19). For instance, individuals with agoraphobia showed increased levels of C-reactive, protein and TNF-α in plasma or serum, respectively, compared to controls (20, 21).

The immune system is closely interconnected, with **neuropeptide signaling** that contributes to the regulation of anxiety and depression states. Neuropeptide Y (NPY) is a bioactive peptide which is abundantly present in the CNS, but also in the postganglionic sympathetic nerve and in immune cells (22–24). It is involved in the regulation of numerous physiological processes, such as energy homeostasis, food intake, emotional behavior, and stress coping, exerting its effects via at least 4 different receptor types (Y1, Y2, Y4, Y5) (25). Since NPY is implicated to have anxiolytic properties and to counteract the anxiogenic effect of corticotropin-releasing hormone (CRH), it is considered crucial for the stress adaptation process (25, 26). Several studies report on altered NPY levels in plasma and/or CSF in posttraumatic stress disorder (PTSD), major depressive disorder (MDD), and chronic stress (27–32). Moreover, NPY is stated to be a modulator of the immune system, influencing immune function both in an autocrine or paracrine manner and as transmitter between the brain stress response and the immune system (23, 24). NPY can induce peripheral or central immune activation or suppression, depending on multiple factors such as the presence of Y receptors and cell types involved (24). Additionally, immunological and other physiological actions of NPY are regulated by its DPPIV-mediated cleavage (24).

CD26/DPPIV is a ubiquitously distributed transmembrane glycoprotein, whose soluble form is present in plasma and which plays an essential role in the immune system, particularly in T cell activation (33). Functioning as a serine protease that cleaves amino-terminal dipeptides with either L-proline or L-alanine at the penultimate position, DPPIV also modulates the bioactivity—mostly by promoting a more rapid degradation—of several chemokines, peptide hormones and neuropeptides, including substance P, glucagon-like peptide 1 (GLP-1), and NPY (34, 35). The anxiolytic effects of NPY are known to be primarily mediated by the interaction of full-length NPY with Y1-receptors, whereas NPY-truncation by DPPIV to NPY_3−36_ is assumed to have an anxiogenic effect due to a higher affinity of NPY_3−36_ to the receptor Y2, and reduced Y1-receptor stimulation (25, 36). Recent animal studies suggest that DPPIV functions as modulator of the hypothalamic-pituitary-adrenal (HPA) axis activity and stress response (37, 38), and altered levels of soluble DPPIV have been reported for different psychiatric disorders (20, 39, 40).

The first aim of the current project was to elucidate peripheral changes in plasma-derived IL-6, TNF-β, NPY, and DPPIV in two common anxiety disorders in child and adolescent psychiatry, SM and SAD, in comparison to a control group (CG). The second aim was to explore the association of these peripheral immune and neuropeptide markers with dimensional measures of BI and severity of anxiety.

## Materials and Methods

### Study Design and Participants

Data were obtained between January, 2015 and June, 2016 at the Department of Child and Adolescent Psychiatry, Psychosomatics and Psychotherapy, Johann Wolfgang Goethe-University, Frankfurt, Germany. The study was conducted in accordance to the Declaration of Helsinki and was approved by the ethics committee of the University Hospital Frankfurt (Ethic approval No. 237/09, amendment No. 3 27.02.2014). Parents and children were informed about the study thoroughly and comprehensively. All parents received written information material. Assent was obtained from the children as well as written informed consent from their parents.

Exclusion criteria for the current study were (1) age below 5 and older than 18 years, (2) other psychiatric diagnoses, except SM and SAD as primary diagnosis respectively and depressive comorbidity, (3) acute sickness, (4) chronic disease of the neural or endocrine system (5) any medication intake, (6) IQ < 85 or (7) not sufficient German language skills. No psychiatric diagnoses were present in the CG.

Diagnosis of SM or SAD according DSM-5 was confirmed by an experienced child and adolescent psychiatrist. In a semi-structured clinical interview with the child/adolescent and the parent present and past psychopathological symptoms were explored. An extensive individual developmental, somatic, and social history was obtained, including information by the child's pediatricians as well as kindergarten or schoolteachers. Body mass index (BMI), puberty status index (41), contraceptives- and smoking status were documented. For the questionnaire-based assessment of BI and anxiety symptoms see below. The child's cognitive abilities were assessed by either the Wechsler Intelligence Scale for Children (WISC IV; (42) or the Snijders-Oomen non-verbal Intelligence test, revised (SON-R 5 $\frac{1}{2}$-17; (43).

### Blood Sampling and Analysis

Blood was collected in a fasting status in the morning using standard EDTA-tubes (1 tube per participant) and BD™ P100 Blood Collection System for Plasma Protein Preservation and BD Vacutainer (1 tube per participant). The processing of the samples was performed immediately to acquire 5–6 × 0.5 ml aliquots of EDTA-Plasma and 5–6 × 0.5 ml aliquots of the “BD P100” Plasma (Plasma stabilized with proteinase inhibitors). Aliquots were stored at −80°C until the analysis. Plasma concentrations of DPPIV, IL-6, TNF-β, and NPY were determined using commercially available ELISA kits (DPP4/CD26 ELISA JP27789, interleukin-6 high sensitivity ELISA BE58061 and TNF-β ELISA BE55011, IBL International, Hamburg, Germany; Human Neuropeptide Y Enzyme Immunoassay Kit EIA-NPY, RayBiotech, Peachtree Corners, GA, USA) according to the manufacturers' instructions. Furthermore, we tried to analyze TNF-α which was not effective due to technical problems. The optical density was measured with a microplate reader (Benchmark Plus™ microplate spectrophotometer, Bio-Rad Laboratories, Hercules, CA, USA); all samples and standards were assayed in duplicate. The intra- and inter-assay coefficients of variation (CV) were ≤ 6% except for the TNF-β ELISA (intra-assay: CV = 8.0%, inter-assay: CV = 10.2%) and the NPY EIA (intra-assay: CV < 10.0%, inter-assay: CV < 15.0%). Within quality control outliers in all neurobiological samples, defined as raw values more than 2.5 *SD*s from group mean, were removed (DPPIV: *n* = 0; IL-6: *n* = 2; TNF-β: *n* = 3; NPY: *n* = 3). Furthermore, samples with high relative variability (CV >0.20) in double measurement were also excluded from analyses (DPPIV: *n* = 0; IL-6: *n* = 2; TNF-β: *n* = 6; NPY: *n* = 0).

### Measures of BI and Psychopathology

**Behavioral inhibition (BI)** as an aspect of child temperament was assessed with the Retrospective Infant Behavioral Inhibition Scale (44). Parents rated their child's behavioral and emotional symptoms in the first 2 years of life retrospectively by 20 items on a five-point Likert-scale. Subscales “Distress,” “Fear,” and “Shyness” as well as a total score for BI were calculated, with higher scores indicating stronger facets of BI. Evaluated in two non-clinical samples, RIBI shows an excellent reliability with an internal consistency of Cronbach's α = 0.91–0.92 (44).

Regarding **anxiety symptoms**, two general psychometric measures and a specific one for social anxiety were used: Anxiety symptoms according to ICD-10 were assessed via the German parent-rating questionnaire FBB-ANZ from the DISYPS-II questionnaires (45). It consists of 33 items on a four-point Likert scale ranging from 0 (“not at all”) to 3 (“very much”). Subscales for Separation Anxiety (10 items), Generalized Anxiety, Social Anxiety, and Specific Phobia (each subscale consisting of 7 items) were extracted as well as a total score. Age- and sex-specific norms were used to transform sum-scores in Stanine-scores (*M* = 5, *SD* = 2), with scores of 8 and 9 indicating clinically relevant psychopathology. In a clinical sample, the FBB-ANZ showed mostly satisfying reliability (internal consistency: Cronbach's α = 0.69–0.93) and good validity (48). The German version of the parent-rating Screen for Child Anxiety Related Emotional Disorders (English original SCARED; (49), FAS-E; (46) is a screening-questionnaire for diverse anxiety disorders (Panic Disorder, Generalized Anxiety Disorder, Separation Anxiety, Social Anxiety) including school avoidance. All subscale scores as well as a total anxiety score were extracted. The questionnaire consists of 41 items rated on a three-point Likert scale, with higher values in the subscale- or total score pointing to more anxiety symptoms. As shown in a clinical sample, the German SCARED parent-version provides good psychometric properties with an internal consistency ranging between α = 0.76 and α = 0.92 and good validity shown by high correlations with CBCL internalizing symptom scores and cross-informant-agreement (50). As measure for symptoms of social anxiety disorder, the German version of the self-rating questionnaire SPAI-C [SPAIK; (47)] was used, consisting of 26 items being rated on a three-point Likert-scale. Cognitive and somatic symptoms of interaction and performance situations are evaluated, resulting in a total score for social anxiety as well as three subscales (“Interaction,” “Performance,” “Cognitive/somatic symptoms”). The SPAIK was proved as reliable (Cronbach's α = 0.92) and valid measure in a clinical sample (51).

For the quantitative assessment of **mutistic behavior** of their child, parents rated the Frankfurt Scale of Selective Mutism (2) in an age-dependent version (FSSM 3–7, 6–11, 12–18). The questionnaire includes a diagnostic scale “General speaking” consisting of 10 dichotomous items (yes/no) and a severity scale consisting of 41 (FSSM 3–7) or 42 items (FSSM 6–11, 12–18) being rated on a five-point Likert scale indicating speaking in different social situations (at school, in public, at home). Besides a total score, subscale scores for General Speaking and Speaking at school, in public and at home were extracted, with higher scores indicating stronger symptom severity. The FSSM showed an excellent internal consistency with Cronbach's Alpha ranging between α = 0.90 and α = 0.98 and good validity shown by a good agreement to the clinician-rated severity (2).

For assessment of depressive symptoms, as typical comorbid symptomatology, the parent-rated FBB-DES of the DISYPS-II questionnaires (45) was used. It comprises 20 items on a four-point-Likert scale (0 = “not at all” to 3 = “very much”), with higher sum-scores indicating more depressive symptoms.

**Figure 1:**
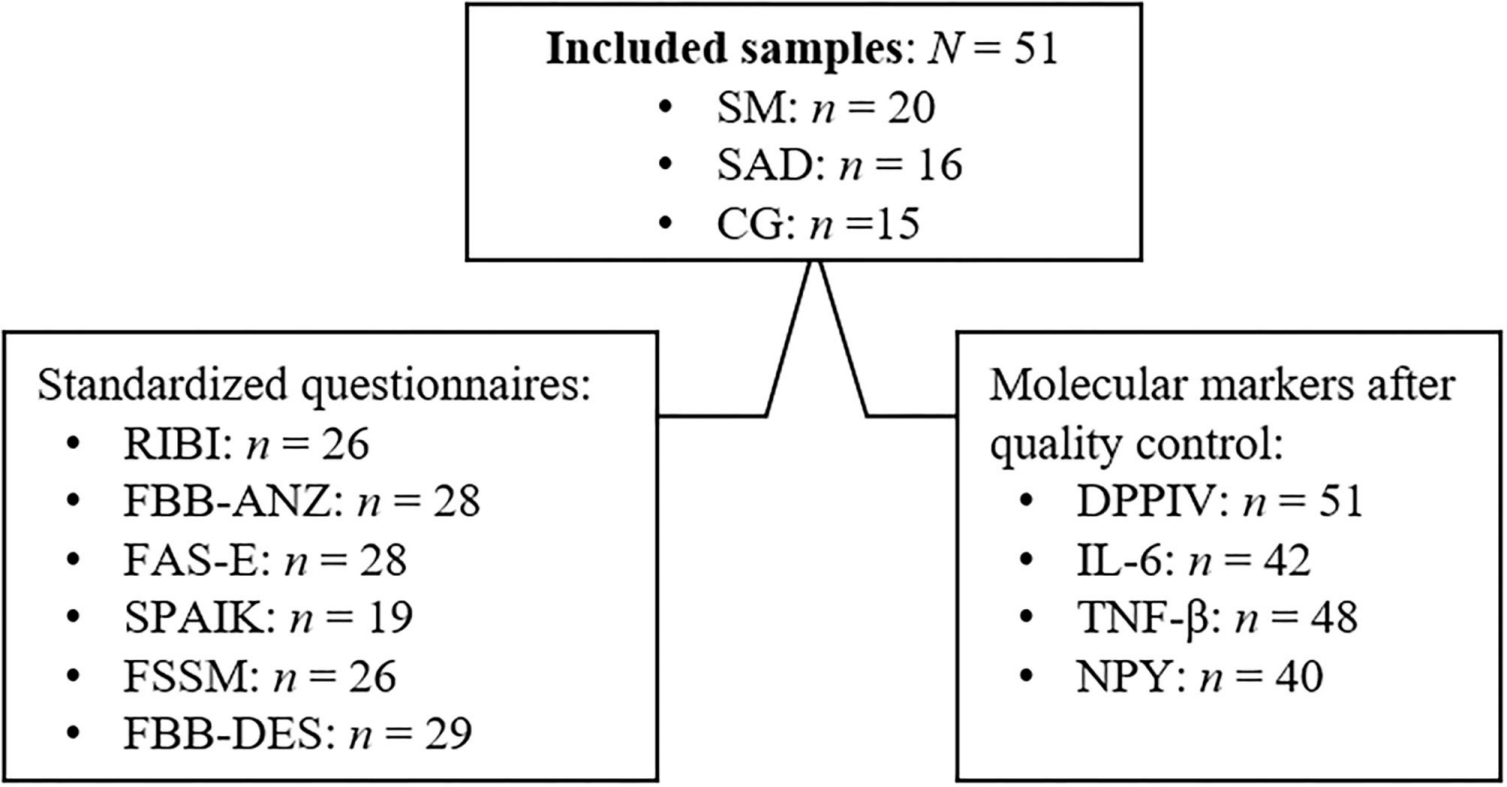
Overview of included samples, available standardized questionnaires and molecular markers. RIBI, Retrospective Infant Behavioral Inhibition Scale (44); FBB-ANZ, parent-rating questionnaire FBB-ANZ from the DISYPS-II questionnaires (45); FAS-E, Fragebogen für Angststörungen—Elternversion, German version of the parent-rating Screen for Child Anxiety Related Disorders (46); SPAIK, Sozialphobie und -angstinventar für Kinder, German version of the self-rating questionnaire SPAI-C (47); FSSM, Frankfurt Scale of Selective Mutism (2); FBB-DES, parent-rating questionnaire FBB-DES from the DISYPS-II questionnaires (45).

An overview of the included samples, available standardized questionnaires, and molecular markers after quality control are depicted in Figure 1.

### Statistical Analysis

Descriptive group differences between SM, SAD, and CG were described by analysis of variance (ANOVAs) or chi-squared tests, as appropriate. Significant main effects in ANOVAs were *post-hoc* tested with *t*-Tests.

In order to reach normal distribution, raw values of blood derived molecular markers were ln-transformed before statistical analyses. Group differences between SM, SAD, and CG in concentrations of molecular markers, BI, and internalizing symptoms were tested with univariate analyses of covariance (ANCOVAs). A significant main effect in an ANCOVA was *post-hoc* studied by *t*-tests.

To examine the association of molecular markers and BI or psychopathology, respectively, multiple regression models with BI measures, anxiety, or mutism symptoms as dependent variable and molecular marker concentrations as independent predictor were applied. Regression analyses were conducted for all children independent of group affiliation.

Age and BMI were consistently added as covariate in ANCOVAs and regression models. Due to a mostly large sample reduction, depressive symptoms as further covariate were only *post-hoc* included in significant models for validating results. Effect sizes for ANOVA and ANCOVA results were computed as partial *η*^2^ (*η*_*p*_^2^), with values *η*_*p*_^2^ < 0.06 interpreted as small 0.06 ≤ *η*_*p*_^2^ < 0.14 as medium and *η*_*p*_^2^ ≥ 0.14 as large effects (52). The effect size detectable with the current sample is *d* = 0.88 (*p* < 0.05, 1-beta = 0.8) for group differences between SM, SAD and CG, and a correlation of *r* = 0.38 can be detected (*p* < 0.05, 1-beta = 0.8) (Power analysis: GPower 3.1.9.4). Bonferroni-adjustment was applied to correct for multiple testing in case of dependent measures of the same trait-/state marker, e.g., subscales in questionnaires. All analyses were carried out with SPSS (Version 21, SPSS, Chicago, USA).

## Results

### Sample Description

The study cohort consisted of *n* = 51 children and adolescents aged between 4.8 and 18.2 years (*M* = 12.2, *SD* = 4.0) with slightly more girls than boys (56.9% girls, χ^2^ = 0.96, *p* = 0.327). According to their principal diagnosis children were assigned to the SM-group, SAD-group or CG, with *n* = 20 SM, *n* = 16 SAD, and *n* =15 CG. All participants agreed to blood sampling for neurobiological marker analyses. After quality control *n* = 40–51 samples remained for analyses (see Blood Sampling and Analysis for more details). The three groups differed in age, with SM-children being younger than the other groups (*F* = 19.44, *p* < 0.001). This is consistent with the typical age-divergent peak of psychopathology, with SM being diagnosed in younger age than SAD (53). Table 1 shows the sample characteristics.

**Table 1:** Sample description.

	**Total sample**	**Groups**			**Group comparison**			
	***(n* = 51)**	**SM *(n* = 20)**	**SAD *(n* = 16)**	**CG *(n* = 15)**	**χ^2^*/F***	***p***	***C/$\eta_{p}^{2}$***	***Group comparison in post hoc tests***
Sex: Girls/Boys	29/22 (56.9 %/43.1 %)	14/6 (70.0 %/30.0 %)	8/8 (50.0 %/50.0 %)	7/8 (46.7 %/53.3 %)	2.35	0.309	0.21	–
Age (years)	12.2 (4.0)	8.9 (3.5)	14.5 (2.5)	14.2 (2.9)	19.44	<0.001	0.45	SM < SP + CG
Weight (kg)	50.3 (21.1)	35.4 (15.6)	61.3 (18.6)	58.6 (19.0)	11.97	<0.001	0.33	SM < SP + CG
Height (cm)	153.9 (23.1)	135.4 (20.8)	166.1 (14.2)	165.6 (16.9)	17.83	<0.001	0.43	SM < SP + CG
BMI	20.1 (3.8)	18.4 (3.4)	21.7 (4.0)	20.5 (3.6)	3.77	0.030	0.14	SM < SP

*Categorial variables are expressed as n (%) and tested with Chi-Squared Tests, effect sizes are expressed as Contingency Coefficients (C). Continuous variables are expressed as Mean (M) with Standard Deviation (SD), group differences are tested with ANOVAs, effect sizes are expressed as Partial Eta Squared ($\eta_{\text{p}}^{2}$). Significant ANOVA results were post-hoc analyzed with t-Tests. SM, selective mutism; SAD, social anxiety disorder; CG, control group*.

Regarding internalizing symptoms, SM-, SAD-group, and CG differed in several diagnostic questionnaires. Regarding anxiety symptoms, differences between groups with large effect size were found for: FBB-ANZ total score (*p* = 0.004, *η*_*p*_^2^ = 0.38) and FBB-ANZ-subscales (*n* = 29; “Generalized anxiety disorder”: *p* = 0.001, *η*_*p*_^2^ = 0.45; “Social anxiety”: *p* = 0.006, *η*_*p*_^2^ = 0.36); for the FAS-E total score (*p* < 0.001, *η*_*p*_^2^ = 0.51) and diverse FAS-E subscales (“Panic disorder,” “Generalized anxiety disorder,” “Social anxiety,” “School avoidance”; *p* < 0.001 to *p* = 0.003, *η*_*p*_^2^ = 0.39–0.59) as well as for the SPAIK total score (*n* = 19, *p* = 0.030, *η*_*p*_^2^ = 0.39) and two SPAIK-subscales (“Performance”: *p* = 0.014, *η*_*p*_^2^ = 0.45; “Cognitive and somatic symptoms”: *p* = 0.003, *η*_*p*_^2^ = 0.57). Descriptively, children with SAD, with SM and controls showed continuously descending symptoms across the anxiety range. In the symptom-specific Scale for Selective Mutism (FSSM), the SM-group showed the highest scores and controls the lowest, with group differences for the total score (*n* = 26, *p* = 0.002, *η*_*p*_^2^ = 0.44) and FSSM-subscales (“General speaking”: *p* = 0.036, *η*_*p*_^2^ = 0.27; “At school”: *p* = 0.007, *η*_*p*_^2^ = 0.38; “In public”: *p* = 0.003, *η*_*p*_^2^ = 0.42). Regarding BI, no group differences were found in RIBI total or subscale scores (*p* = 0.125–0.839, *η*_*p*_^2^ = 0.02–0.18). Regarding depressive symptoms (*n* = 29), as a frequent comorbid psychopathology in anxiety disorders, the SAD-group showed higher scores than SM or CG (*p* = 0.005, *η*_*p*_^2^ = 0.35).

Due to group differences, age and BMI were consistently included as covariates in statistical analyses. To avoid confounding by depressive symptoms but prevent large sample reduction, the respective score was only *post-hoc* included as covariate in the different ANCOVA and regression models to validate results.

### Case-control Differences in Neuropeptide and Immune Markers

SM-, SAD-group and CG did not differ in any parameter as shown in Table 2. The *post-hoc* inclusion of depressive symptoms as further covariate, resulting in sample reduction (*n* = 21–29), did not change non-significant results (*p* = 0.122–0.944).

**Table 2:** Descriptive characteristics of immune and neuropeptide markers and group comparison.

	**Total sample**		**SM**		**SAD**		**CG**		**Group differences**^**a**^		
	***n***	***M (SD)***	***n***	***M (SD)***	***n***	***M (SD)***	***n***	***M (SD)***	***F***	***p***	**$\eta_{\text{p}}^{2}$**
DPPIV (ng/mL)	51	382.1 (99.6)	20	431.2 (90.5)	16	338.3 (95.9)	15	363.3 (92.1)	0.69	0.505	0.03
IL-6 (pg/mL)	42	1.3 (1.1)	16	1.3 (1.0)	15	1.3 (1.3)	11	1.3 (1.0)	0.19	0.826	0.01
NPY (ng/mL)	48	9.4 (4.3)	19	9.3 (3.7)	15	9.2 (2.6)	14	9.8 (6.3)	1.79	0.179	0.08
TNF-β (pg/mL)	40	74.5 (71.3)	16	80.4 (81.9)	14	73.3 (77.2)	10	66.9 (46.1)	0.24	0.790	0.01

*Continuous variables are expressed as Mean (M) with Standard Deviation (SD). Group differences are tested with ANCOVAs, effect sizes are expressed as Partial Eta Squared ($\eta_{\text{p}}^{2}$). SM, selective mutism; SAD, social anxiety disorder; CG, controls*.

a *ANCOVAs are based on ln-transformed raw-values with age and BMI included as covariates*.

### Associations of Neuropeptide and Immune Markers With Anxiety and Mutism Symptoms

Controlling for age and BMI, in independent regression models, DPPIV levels predicted the FAS-E subscale scores for Panic disorder (*n* = 28; regression model: $R_{corr}^{2}$ = 0.31, *F* = 6.28, *p* = 0.003; DPPIV: β = −0.48, 95%-CI for *B* = [−11,19; −1,49], *p* = 0.013) and for School avoidance (*n* = 28; regression model: $R_{corr}^{2}$ = 0.32, *F* = 5.27, *p* = 0.006; DPPIV: β = −0.42, 95%-CI for *B* = [−7.52; −0.41], *p* = 0.030) as well as the FAS-E total score (regression model: $R_{corr}^{2}$ = 0.30, *F* = 4.79, *p* = 0.009; DPPIV: β = −0.52, 95%-CI for *B* = [−49.99; −7.51], *p* = 0.010).

Adding depressive symptoms as further covariate led to a non-significance in the prediction of DPPIV for the FAS-E subscale School avoidance (*n* = 27; regression model: $R_{corr}^{2}$ = 0.31, *F* = 3.90, *p* = 0.015; DPPIV: β = −0.34, 95%-CI for *B* = [−7.29; 0.55], *p* = 0.550). However, the results were strengthened for FAS-E total score (regression model: $R_{corr}^{2}$ = 0.64, *F* = 12.42, *p* < 0.001; DPPIV: β = −0.51, 95%-CI for *B* = [−45.79; −12.33], *p* = 0.002) and FAS-E subscale Panic disorder (*n* = 27; regression model: $R_{corr}^{2}$ = 0.67, *F* = 15.84, *p* < 0.001; DPPIV: β = −0.48, 95%-CI for *B* = [−10,35; −3,00], *p* = 0.001), with both withstanding correction for multiple testing (FAS-E, 6 tests, α-level = 0.008). Lower DPPIV levels predicted more anxiety.

No regression model for DPPIV predicting FBB-ANZ, SPAIK or FSSM reached *p* < 0.05. Furthermore, we did not find any significant model for the other investigated neuropeptide and immune markers IL-6, TNF-β, and NPY.

**Figure 2:**
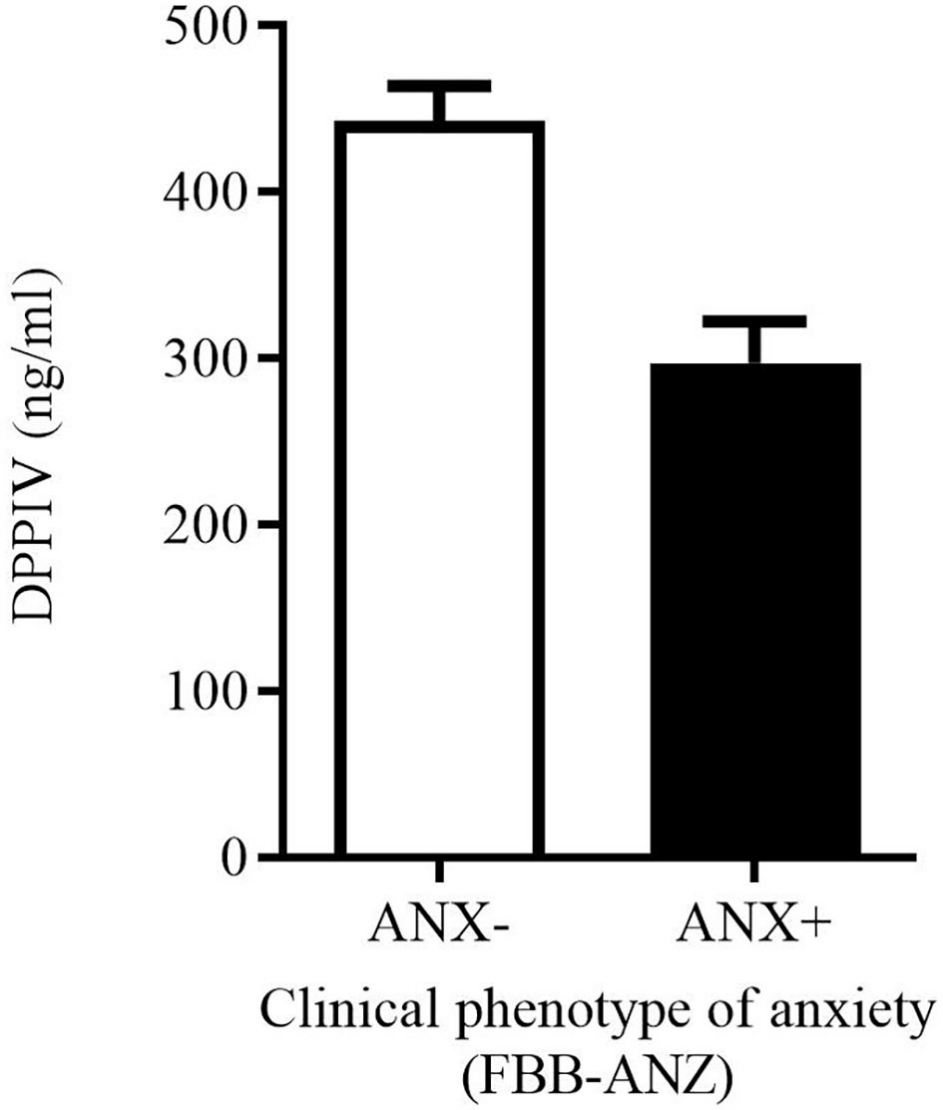
Group difference in DPPIV levels between children and youth with pathological anxiety (ANX+; *n* = 10) vs. non-pathological anxiety (ANX-; *n* = 17) according to the FBB-ANZ (cut-off: Stanine ≥ 8).

Due to the consistent negative associations between DPPIV levels and anxiety symptoms spanning several psychometric measures, but not reaching significance in regression models with exception of the FAS-E, we conducted an exploratory group comparison. Groups were categorized for pathological vs. non-pathological anxiety psychopathology in FBB-ANZ (cut-off: Stanine ≥ 8, *n* = 10 vs. Stanine < 8, *n* = 17). Controlling for age and BMI, both groups differed in DPPIV-levels (*n* = 27; *p* = 0.017, *η*_*p*_^2^ = 0.22): children with a clinical phenotype of anxiety showed reduced DPPIV levels (clinical anxiety: *M* = 297.2, *SD* = 79.3; non-clinical: *M* = 443.0, *SD* = 86.4) as shown in Figure 2. Controlling additionally for depressive symptoms (which did not reduce sample size here), strengthens the result in form of a large group difference (*p* = 0.001, *η*_*p*_^2^ = 0.38).

### Associations of Neuropeptide and Immune Markers With BI

Controlling for age and BMI, TNF-β predicted the “Shyness” -score in the RIBI (*n* = 20; regression model: $R_{corr}^{2}$ = 0.31, *F* = 3.77, *p* = 0.032; TNF-β: β = 0.57, 95%-CI for *B* = [1.04; 6.83], *p* = 0.011), surviving correction for multiple testing (RIBI, 4 tests, α-level = 0.0125). Higher TNF-β-levels predicted more parent-rated shyness. However, adding depressive symptoms as further covariate, resulted in a non-significant regression model (*n* = 18; regression model: $R_{corr}^{2}$ = 0.11, *F* = 1.50, *p* = 0.259). No further regression model reached *p* < 0.05.

## Discussion

The current study investigated neuropeptide and immune markers in peripheral blood of children and adolescents diagnosed with SM and SAD, as well as the association of these proteomic markers with dimensional measures of BI, anxiety and mutistic behavior—independently of the psychiatric diagnosis. Comparing the groups, no differences in any neuropeptide and immune marker could be found, neither between SM and SAD nor between patients with SM and SAD and controls without a psychiatric diagnosis. However, looking at the clinical phenotype irrespectively of diagnosis yielded some interesting findings that may extend our knowledge about the underlying mechanisms of anxiety. Spanning diverse psychometric measures, more anxiety symptoms were consistently associated with lower DPPIV-levels; specifically, DPPIV levels significantly predicted child anxiety in the parent-rated FAS-E, controlled for relevant confounders. The negative association was underlined by a group difference in DPPIV-levels found between children with a clinical phenotype of anxiety vs. normal anxiety levels.

Several other studies found an inverse correlation between circulating DPPIV levels and phobic anxiety as well as depression (20, 39, 54). Wagner et al. (35) showed the decline of soluble DPPIV in acute depression that could be reversed upon anti-depressive treatment. DPPIV-activity was furthermore demonstrated to be decreased in patients with unipolar depression (40). These studies were performed in adult patient groups, so to the best of our knowledge, this is the first time that the inverse correlation between DPPIV and internalizing symptoms of anxiety was shown in children and adolescents. However, the molecular mechanisms causative of this relationship between soluble DPPIV levels and anxiety symptoms need further investigation. Emanuele et al. (20) hypothesized that lowered circulating DPPIV levels in subjects with higher anxiety scores could reflect a genetic profile linked to vulnerability to anxiety, and that the soluble DPPIV plasma concentration might then serve as a risk marker. Wagner et al. (35) assumed an immunological link between reduced soluble DPPIV and symptoms of depression, since immune cells and bone marrow have been identified as two sources of soluble DPPIV (35).

Several stress-related neuropeptides implicated in anxiety, depression and schizophrenia are substrates of DPPIV, including NPY (35). Considering the hypothesis that a shift from anxiolytic to anxiogenic effects of NPY is promoted by DPPIV activity—inducing its rapid degradation and a switch in Y-receptor affinity—our findings of lowered DPPIV and unaltered NPY levels in anxiety patients seem somewhat contradictory. However, it is important to note that anxiety-related effects of NPY are exerted in the brain, whereas we assessed both soluble DPPIV and NPY levels in plasma. A recent meta-analysis (27) determined that both plasma and CSF NPY levels are reduced in PTSD; yet there are also studies reporting on unaltered plasma NPY levels in PTSD (55, 56), and Baker et al. (57) provided evidence of weak correlation between CSF and plasma NPY pools. In MDD, meta-analysis revealed that plasma, but not CSF NPY levels were significantly lowered (27). Interestingly, Wagner et al. (35) report that compared to healthy controls, the time-dependent hydrolysis of NPY in sera of depressed patients was not altered, in spite of lowered DPPIV concentrations. They conclude that the degradation of NPY in blood circulation is complex as it comprises not only soluble serum enzymes, but also membrane-bound peptidases in/on endothelial cells and blood cells, and that it may be regulated by immune responses of various disease conditions (35). Finally, peripheral NPY is a sympathetic neurotransmitter and potent vasoconstrictor that is released during sympathetic nerve stimulation, preferentially during prolonged and/or intense stress (58, 59) and chronic stress was reported be associated with significantly increased plasma NPY levels (27). Overall, findings on plasma NPY concentrations and their relation to circulating DPPIV in the context of anxiety disorders must be interpreted with caution, and our present results need to be confirmed in larger follow-up studies.

Supporting the view of psychiatric disorders as systemic diseases with molecular alterations in both the brain and peripheral tissues, changes in plasma concentrations of molecular targets and their corresponding protein networks have been identified for several psychiatric disorders, such as major depression, bipolar disorder and schizophrenia (e.g., (60–62). When validated, biomarkers can provide information on disease risk and disease severity as well as help to predict the response of an individual to a given intervention and thus individualizing and optimizing the choice of therapy. Last but not least, a better understanding of the molecular pathways associated with mental disorders could significantly contribute to our knowledge of disease pathophysiology, with the potential to transform the disease classifications/diagnosis and consequently the treatment, as the NIH Project on Research Domain Criteria (RDoC) hopes to achieve. The current associations of DPPIV with anxiety independently of the child's diagnose challenge the discrete classification and point to the dimensional characteristic of symptomatology and their etiology. This is in line with a meta-analysis (63) showing that SM is diagnosed in 80% in combination with anxiety disorders indicating common biological basis of these disorders. However, further research is needed to investigate the etiology of SM and SAD in order to clarify the similarities and differences.

### Limitations

First of all, the results of the current pilot study must be interpreted as preliminary due to small sample size. Because of the wide age range and the naturalistic diagnostic procedure regarding questionnaires (focused on main symptoms), the psychometric measures were not consistently available for all children/adolescents resulting in varying sample size dependent on measure. Quality control in molecular analyses and the inclusion of relevant covariates (e.g., depressive symptoms) in analyses resulted in further sample reduction. Especially, group analyses of psychometric measures with sample sizes ranging between 4 and 15 children must be interpreted cautiously; group analyses of molecular markers with groups ranging between *n* = 10–20 children can be seen as more valid but must be replicated in larger cohorts. Furthermore, due to sample size, sex-specific analyses could not be carried out. Especially due to the well-known sex-difference in the prevalence of anxiety disorders, this is recommended for future research in order to give a more differentiated insight into specific, divergent processes between girls and boys. Secondly, the current study used a cross-sectional design, thus enabling only correlational and not causative statements. Future studies with a prospective longitudinal design should reevaluate the current preliminary findings and give more insights into the hypothesis of SM and SAD having a common biological basis by analyzing developmental processes.

## Conclusions

The association of neuropeptide and immune markers with measures of BI, anxiety and mutistic behavior in children and adolescents diagnosed with SM and SAD was analyzed here for the first time. While molecular markers were associated with a state marker (anxiety), grouping by diagnoses of SM and SAD did not result in divergent molecular patterns. This underlines the importance of investigating the pathophysiology of symptoms independent of diagnoses following the RDoC-initiative. Larger and prospective studies are needed to replicate and validate the current results and to expand the knowledge about the causative core biological mechanisms underlying anxiety. This might help better understand and differentiate the currently symptom-based diagnoses of SM and SAD.

## Data Availability Statement

The raw data supporting the conclusions of this article will be made available by the authors, without undue reservation.

## Ethics Statement

The studies involving human participants were reviewed and approved by Ethics committee of the University Hospital Frankfurt (Ethic approval No. 237/09, amendment No. 3 27.02.2014). Written informed consent to participate in this study was provided by the participants' legal guardian/next of kin.

## Author Contributions

YG and CF initiated and designed the study and supervised the clinical data acquisition. YG, VS, AP, AE, RW, and CF analyzed the data and/or interpreted the results. OK helped to conceptualize the clinical diagnostic. RW established laboratory methods, i.e., preparation of the blood samples and protocols. SH was part of the conceptualization-team of the study, especially regarding the role of immune system, and DPPIV/NPY system in anxiety disorders. VR participated in the conceptualization of the study. YG, VS, AP, SH, and VR wrote and prepared the manuscript. All authors reviewed the manuscript.

## Conflict of Interest

The authors declare that the research was conducted in the absence of any commercial or financial relationships that could be construed as a potential conflict of interest.
